# Free Flap Reconstruction of Xylazine‐Associated Wounds: A Retrospective Review

**DOI:** 10.1002/micr.70189

**Published:** 2026-03-05

**Authors:** Alan T. Makhoul, Carrie Z. Morales, Elizabeth B. Card, Matthew A. Goldshore, Jon B. Morris, L. Scott Levin, Jason D. Wink, John P. Fischer, Ines C. Lin, Stephen J. Kovach

**Affiliations:** ^1^ Division of Plastic Surgery, Department of Surgery University of Pennsylvania Philadelphia Pennsylvania USA; ^2^ Center for Surgical Health, University of Pennsylvania Philadelphia Pennsylvania USA; ^3^ Department of Surgery Children's Hospital of Philadelphia Philadelphia Pennsylvania USA; ^4^ Department of Surgery University of Pennsylvania Philadelphia Pennsylvania USA; ^5^ Department of Orthopaedic Surgery University of Pennsylvania Philadelphia Pennsylvania USA

**Keywords:** injection drug, limb salvage, substance use, wound, xylazine

## Abstract

**Background:**

Xylazine is a veterinary sedative that is added to illicit fentanyl to enhance its effects. Xylazine‐associated wounds differ from those found in patients who inject other drugs. They are larger and frequently involve deeper structures, such as bone. The outcomes of reconstruction using free tissue transfer are not well understood.

**Methods:**

All consecutive free flap reconstructions of xylazine‐associated wounds at a tertiary care center in the northeast US between January 2021 and December 2024 were retrospectively reviewed. Data were stored in a HIPAA‐compliant REDCap database.

**Results:**

Eleven free flap reconstructions were performed among 10 patients. Median age was 34 years (IQR: 31–38), all were White, 20% were Hispanic, and 90% were female. Median BMI was 20.9 (IQR: 19.9–39.3). Comorbidities included HCV (70%) and active tobacco smoking (60%). Wounds were located on the neck (9.1%), chest (18.2%), upper extremity (45.5%), and hand (27.3%). 81.8% presented with exposed bone. Median debridements were two (IQR 1–3). Free flaps included: four anterolateral thigh (36.4%), three gracilis (27.3%), one rectus abdominis (9.1%), one scapular (9.1%), one latissimus dorsi (9.1%), and one lateral arm (9.1%) flap. Two patients were discharged against medical advice (18.2%). Median follow‐up was 364 days (IQR: 287–710). All flaps were viable at 3 weeks. Roughly half of patients (45.5%) continued to use injection drugs after reconstruction. Early complications included: one venous congestion requiring exploration and one partial flap dehiscence. Late complications included two surgical site infections and two wound recurrences due to continued xylazine use resulting in trans‐humeral amputation (40% of those who continued to inject drugs).

**Conclusion:**

Free tissue transfer can effectively reconstruct xylazine‐associated wounds and is necessary for limb salvage in patients with exposed bone. All flaps were viable at 3 weeks. Late wound recurrence due to continued xylazine use is associated with poor outcomes, including amputation. Abstinence from injection drug use is critical to optimizing the chances of recovery, and a multidisciplinary approach is essential.

## 1 Introduction

Xylazine is a veterinary tranquilizer that is used as an additive in the illicit fentanyl supply. It is an alpha‐adrenergic receptor agonist that decreases the release of norepinephrine and dopamine in the central nervous system (Rubin 2023). Xylazine is not approved for human use and is thought to prolong the effects of the opioid. It is most prevalent in the northeast US, though it has been detected by the US Drug Enforcement Agency in 48 states (DEA (United States Drug Enforcement Agency), n.d.; Gupta et al. 2023). In 2022, 91% of fentanyl samples tested by the Philadelphia Department of Health contained xylazine (Drug Checking Quarterly Report (Q3 2022) 2022).

When injected subcutaneously, xylazine produces large, complex soft tissue wounds that differ from those historically seen in patients who inject other drugs. Unlike skin lesions associated with heroin that often begin as an abscess or cellulitis, xylazine‐associated wounds (XAWs) do not typically start as an infection (Yang et al. 2025). XAWs are larger, often involve deep structures such as bone, and frequently present with eschar or devitalized tissue (Lutz et al. 2025). XAWs most often occur on the extremities at the injection site, though they have been reported on all parts of the body, including the scalp and chest (Pomerantz et al. 2025; O'Neil and Kovach 2023). Limb loss is common among patients with XAWs, with some reporting amputation rates as high as 13% (Tosti et al. 2024; Weir et al. 2024).

Reconstruction of XAWs typically focuses on substance use rehabilitation and local wound care to prepare the wound bed for definitive skin grafting. Systemic illness, such as sepsis, resulting from XAWs is rare (Johnsen et al. 2025). Most patients present with a chronic wound that can be optimized for reconstruction through autolytic debridement and non‐adherent dressing changes (Lutz et al. 2025). Multiple algorithms have been proposed for the surgical management of XAWs (Johnsen et al. 2025; Retrouvey et al. 2024).

Some patients, however, present with a need for stable soft tissue coverage of exposed bone or critical structures, such as the airway. Patients with a pathologic fracture and concurrent need for soft tissue resurfacing represent a particularly challenging reconstructive problem. In these patients, free flap reconstruction is the only option to avoid amputation, though the outcomes of free tissue transfer in this setting are not well understood. In this study, we review our experience with free flap reconstruction of XAWs to better understand the utility.

## 2 Methods

A retrospective review of all consecutive free flap reconstructions of xylazine‐associated wounds at a tertiary care center in the northeast US between January 2021 and December 2024 was conducted. Institutional review board exemption was obtained (#855779). Patients provided consent to the use of their clinical photographs. Chart review was completed in July 2025 to allow a minimum six‐month follow‐up period.

Patients were identified through report generation in epic systems based on opioid use disorder ICD‐10 codes and free flap CPT codes 15756, 15757, and 15758. Charts were manually reviewed for xylazine‐associated wounds, and xylazine use history was determined by patient report and assessment of clinical photographs. In Philadelphia during this period, greater than 90% of fentanyl samples contained xylazine (Drug Checking Quarterly Report (Q3 2022) 2022; Lutz et al. 2025). Continuous and categorical variables were recorded in a deidentified REDCap database. One patient underwent bilateral upper extremity free flaps, and these were recorded separately. Substance use history was determined by patient report and urine drug screen testing. Follow‐up was recorded as outpatient or inpatient encounters.

## 3 Results

Eleven free flap reconstructions were performed among 10 patients. Median age was 34 years (IQR: 31–38), all were White, 20% were Hispanic, and 90% were female (Table 1). Median BMI was 20.9 kg/m^2^ (IQR: 19.9–39.3). Comorbidities included HCV (70%) and active tobacco smoking (60%). None injected 1 week pre‐operatively, four injected in the preceding month, and five in the preceding 1–6 months. 90% reported reliable housing, and 80% were unemployed.

**TABLE 1:** Patient characteristics.

	*N* (%)
Median age in years (IQR)	34 (31–38)
Race	
White	10 (100)
Ethnicity	
Hispanic or Latino	2 (20)
Not Hispanic or Latino	8 (80)
Sex	
Male	1 (10)
Female	9 (90)
Median BMI (IQR)	20.9 (19.9–39.3)
Comorbidities	
Diabetes	0 (0)
HCV	7 (70)
HIV	0 (0)
Hypercoagulable disorders	0 (0)
Tobacco history	
Current smoker	6 (60)
Quit smoking	2 (20)
Never smoker	2 (20)
Reliable housing	
Yes	9 (90)
No	1 (10)
Employed	
Yes	2 (20)
No	8 (80)
Injection drug history at time of free flap	
Within last week	0 (0)
Within last month	4 (36.4)
1–3 months	2 (18.2)
3–6 months	3 (27.3)
6–12 months	0 (0)
1+ years ago	1 (9.1)
Unknown	1 (9.1)
Surgeon	
A	3 (27.3)
B	4 (36.4)
C	3 (27.3)
D	1 (9.1)

**FIGURE 1:**
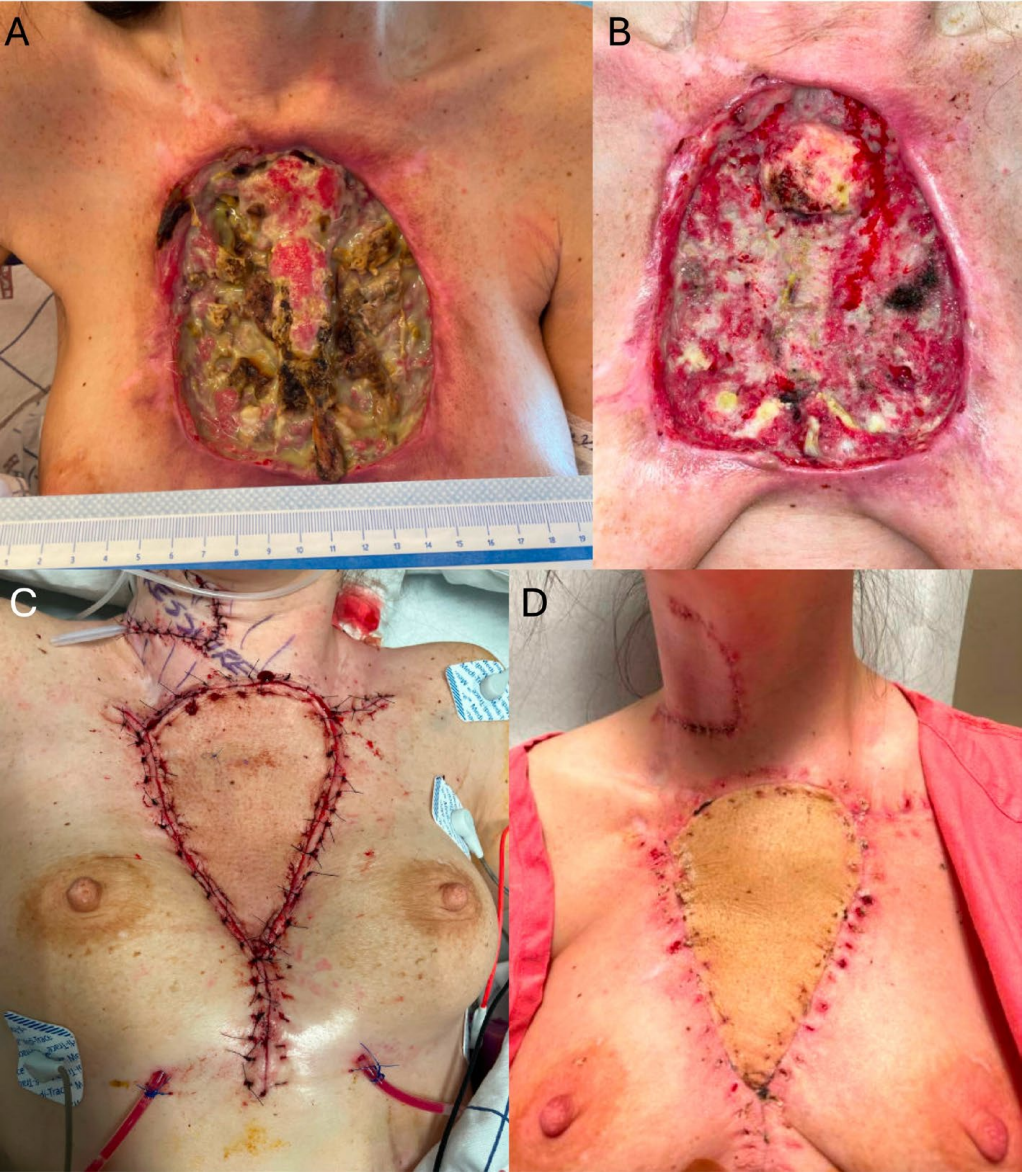
(A) 32‐year‐old female who presented to the emergency department with a large anterior chest xylazine‐associated wound of 2 months duration. (B) Wound after debridement of necrotic bilateral clavicles, bilateral sternoclavicular joints, sternal body, and anterior ribs. (C) Reconstruction using right anterolateral thigh free flap with arterial anastomosis to the superior thyroid artery and venous anastomosis to the facial vein. (D) Flap viable at three‐week outpatient follow‐up.

Wounds were located on the neck (9.1%), chest (18.2%), upper extremity (45.5%), and hand (27.3%) (Table 2). Most upper extremity and hand wounds (75%) involved the extensor surfaces. Nine (81.8%) presented with exposed bone. One (9.1%) presented with an exposed airway. The median number of debridements prior to free flap reconstruction was two (IQR: 1–3). Osteomyelitis was managed with bone burring in four patients (36.4%) and bone resection in six patients (54.5%) (Figure 1). Two wounds were temporized with skin substitutes prior to free flap, and one was treated unsuccessfully with skin grafting.

**TABLE 2:** Free flap reconstruction outcomes.

	*N* (%)
Wound location	
Neck	1 (9.1)
Chest	2 (18.2)
Right upper extremity	3 (27.3)
Left upper extremity	2 (18.2)
Right hand	1 (9.1)
Left hand	2 (18.2)
Structures involved	
Skin, subcutaneous tissue only	1 (9.1)
Bone exposed	9 (81.8)
Airway exposed	1 (9.1)
Median OR debridements prior to free flap (IQR)	2 (1–3)
Osteomyelitis management	
Antibiotics	9 (81.8)
Bone burring	4 (36.4)
Bone resection	6 (54.5)
No history of osteomyelitis	1 (9.1)
Reconstruction prior to free flap	
Skin substitute	2 (18.2)
Skin graft	1 (9.1)
Local flap	0 (0)
Free flap type	
Anterolateral thigh	4 (36.4)
Gracilis	3 (27.3)
Rectus abdominus	1 (9.1)
Scapula	1 (9.1)
Latissimus dorsi	1 (9.1)
Lateral arm	1 (9.1)
Flap composition	
Fasciocutaneous	6 (54.5)
Musculocutaneous	2 (18.2)
Muscle only	3 (27.3)
Arterial anastomosis	
End‐to‐end	2 (18.2)
End‐to‐side	9 (81.8)
Venous anastomosis	
One vein	5 (45.5)
Two veins	6 (54.5)
Median post‐operative hospitalization in days (IQR)	7 (6–13)
Discharge AMA post‐operatively	
Yes	2 (18.2)
No	9 (81.8)
Median follow‐up in days (IQR)	364 (287–710)
Flap outcome	
Full survival	11 (100)
Partial survival	0 (0)
Total loss	0 (0)
Early complications (< 3 weeks post‐operatively)	
Venous congestion	1 (9.1)
Partial flap dehiscence	1 (9.1)
Late complications (> 3 weeks post‐operatively)	
Surgical site infection	2 (18.2)
Wound recurrence due to xylazine	2 (18.2)
Re‐operations	
Exploration	1 (9.1)
Sequestrectomy of radius	1 (9.1)
Wrist denervation	1 (9.1)
Trans‐humeral amputation	2 (18.2)
Injection drug use after free flap	
Yes	5 (45.5)
No	6 (54.5)

Abbreviation: AMA, against medical advice.

Free flaps performed included: four anterolateral thigh (ALT) (36.4%), three gracilis (27.3%), one rectus abdominis (9.1%), one scapular (9.1%), one latissimus dorsi (9.1%) and one lateral arm (9.1%) (Table 3). Six flaps were fasciocutaneous (54.5%), two were musculocutaneous (18.2%), and three were muscle‐only (27.3%). For six flaps (54.5%), two venous anastomoses were performed. Most arterial anastomoses (81.8%) were performed end‐to‐side. The median post‐operative hospitalization was 7 days (IQR: 6–13). Two patients were discharged against medical advice (AMA) (18.2%). The median follow‐up was 364 days (IQR: 287–710). All flaps were viable, and limb salvage was achieved at 3 weeks post‐operatively.

**TABLE 3:** Individual case summary and outcomes.

	Wound location	Most recent IVDU prior to free flap	Prior operative debridements	Prior skin graft or substitute	Free flap (composition)	Post‐operative hospitalization, in days	IVDU after free flap	Complications	Time to most recent follow‐up, in days	Outcome
1	Dorsal hand	3–6 months prior	1	1	Lateral arm (fasciocutaneous)	6	No	Minor flap dehiscence observed POD 12 managed with local wound care	988	Fully healed
2	Volar forearm	Prior month	5	0	Anterolateral thigh (fasciocutaneous)	6	Yes	Xylazine wound recurrence observed POD 388	687	Trans‐humeral amputation
3	Volar arm	Unknown	2	0	Rectus abdominis (musculocutaneous)	5; discharged AMA	No	None	284	Fully healed
4	Dorsal forearm	3–6 months prior	9	0	Scapula (fasciocutaneous)	6	No	Osteomyelitis of radius observed POD 123 managed with sequestrectomy	291	Fully healed
5	Dorsal forearm	1–3 months prior	2	0	Latissimus (musculocutaneous)	18; discharged AMA	Yes	Venous congestion on POD 1 requiring exploration; osteomyelitis of radius observed POD 127 managed with antibiotics	282	Fully healed
6	Volar and dorsal forearm	1–3 months prior	2	0	Gracilis (muscle only)	7	Yes	Xylazine wound recurrence observed POD 300	472	Trans‐humeral amputation
7	Dorsal hand	3–6 months prior	1	0	Gracilis (muscle only)	8	No	None	733	Fully healed; wrist denervation for chronic pain
8	Dorsal hand	> 1 year prior	3	1	Gracilis (muscle only)	8	No	None	364	Fully healed
9	Chest	Prior month	3	1	Anterolateral thigh (fasciocutaneous)	46	Yes	None	784	Fully healed
10	Chest	Prior month	2	0	Anterolateral thigh (fasciocutaneous)	4	No	None	108	Fully healed
11	Neck	Prior month	0	0	Anterolateral thigh (fasciocutaneous)	44	Yes	None	305	Fully healed

Abbreviations: AMA, against medical advice; IVDU, intravenous drug use; POD, post‐operative day.

**FIGURE 2:**
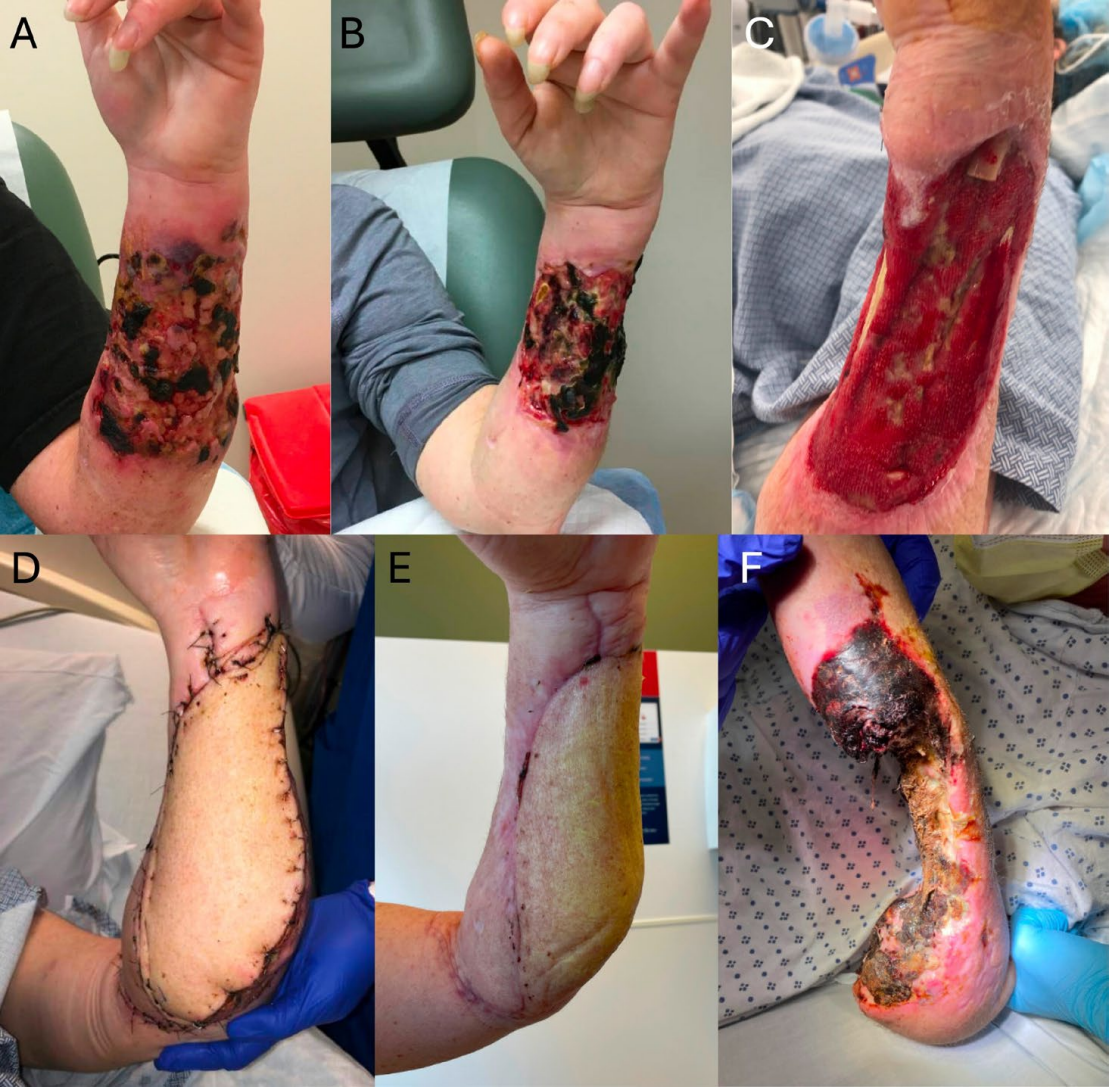
(A) 39‐year‐old female who presented to clinic with a chronic left forearm xylazine‐associated wound. (B) Follow‐up after 1 month of autolytic debridement with medical honey. (C) Wound after resection of 11 cm of ulna and debridement of flexor carpi radialis, flexor digitorum superficialis, flexor carpi ulnaris, and extensor carpi ulnaris tendons. (D) Reconstruction using right anterolateral thigh free flap with end‐to‐side arterial anastomosis to the brachial artery and two venous anastomoses to venae comitantes. (E) Flap viable at 6‐week outpatient follow‐up. (F) Presentation on post‐operative day 388 after reconstruction with recurrent xylazine‐associated wound requiring trans‐humeral amputation.

Early complications (occurring in the first 3 weeks post‐operatively) included: one venous congestion due to pedicle twisting requiring exploration post‐operative day one and one partial flap dehiscence treated with local wound care. Late complications included two recurrences of osteomyelitis and two wound recurrences observed on post‐operative days 388 and 300 due to continued xylazine use resulting in trans‐humeral amputation (Figure 2). Roughly half of patients (45.5%) continued to use injection drugs after reconstruction.

## 4 Discussion

**FIGURE 3:**
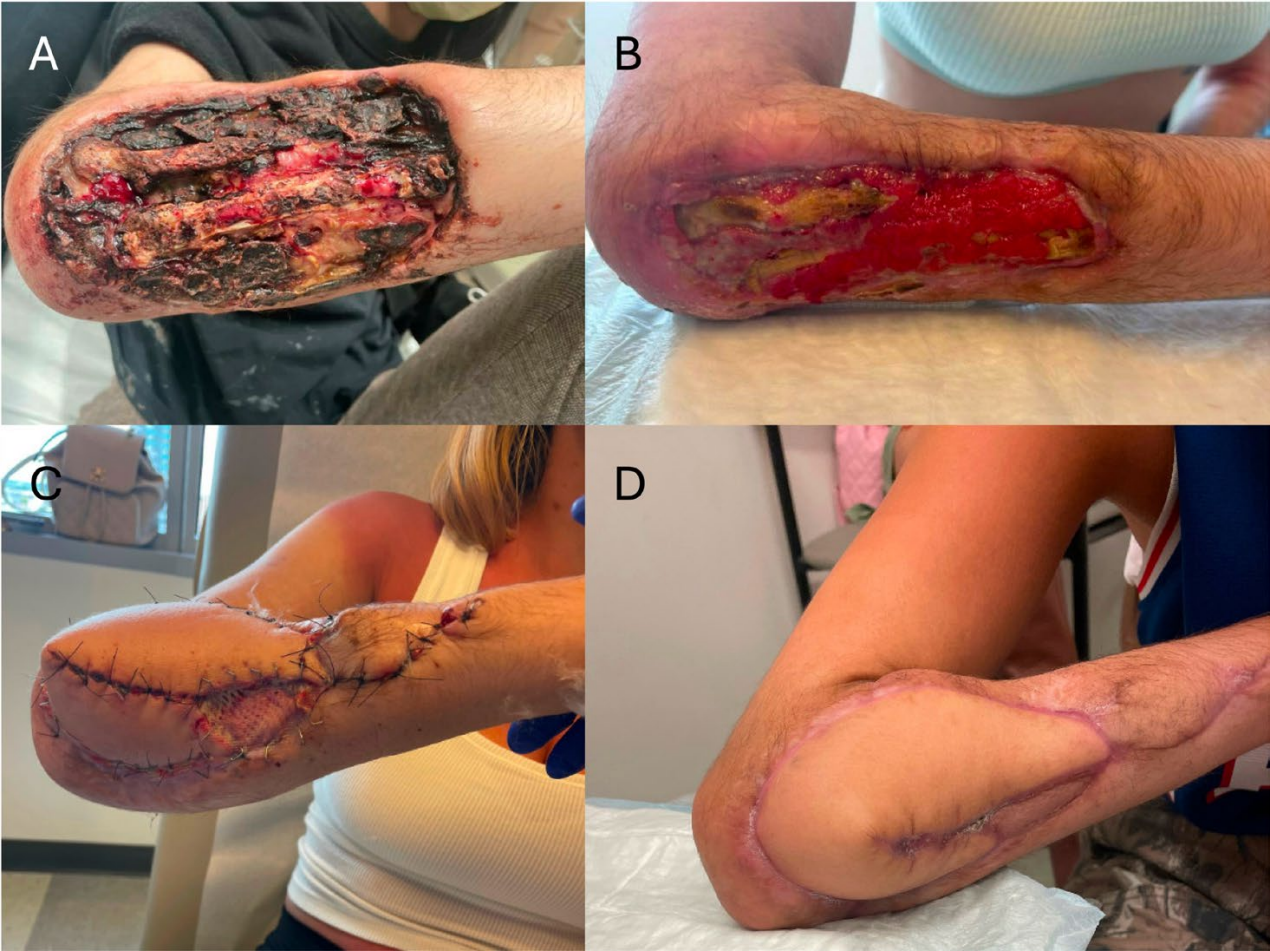
(A) 30‐year‐old female who presented to the emergency department with a 3‐year history of right dorsal forearm xylazine‐associated wound. (B) Clinic presentation after 2 months of local wound care using dilute sodium hypochlorite solution. (C) Ten days after reconstruction using left scapular free flap with end‐to‐side arterial anastomosis to the brachial artery and two venous anastomoses to venae comitantes. One small split‐thickness skin graft was also used. (D) Flap healed at three‐month outpatient follow‐up.

Free tissue transfer is an effective reconstructive option for patients presenting with exposed bone or critical structures in the setting of a xylazine‐associated wound (XAW) (Figure 3). Fasciocutaneous, musculocutaneous, and muscle‐only free flaps were performed for soft tissue resurfacing and all were viable at 3 weeks. Other than comorbidities related to substance use disorder, patients presenting with XAWs were young and healthy. Reconstruction offers these patients a substantial benefit in quality‐adjusted life years and an opportunity for limb salvage.

Adequate recipient site preparation is critical to preventing surgical site infection and post‐operative complications. Although XAWs rarely result in systemic illness, they are typically colonized, most often by methicillin‐resistant *Staphylococcus aureus* (MRSA) or group A streptococcus (GAS) (Yang et al. 2025). In cases of infection, trimethoprim/sulfamethoxazole to cover MRSA plus cephalexin to cover GAS, is a typical empiric oral antibiotic regimen (Yang et al. 2025). A staged approach to debridement is typically necessary to optimize the wound. In our series, patients underwent a median two operative debridements prior to free tissue transfer. Necrotic skin, fat, and muscle must be removed. When present, osteomyelitic bone should be burred or resected (Fallahi et al. 2025). Two patients underwent skin substitute placement to temporize their wounds and act as a soft tissue scaffold. One benefit of free flap reconstruction over skin substitutes is the recruitment of healthy muscle and soft tissue to the site of chronic osteomyelitis helps facilitate resolution of infection more effectively.

Microsurgical anastomosis should be performed outside the zone of injury, which may necessitate vein grafting given the extent of some wounds. CT angiogram of the affected site is helpful to ensure patent recipient vessels, as well as to rule out abscess or pathologic fracture. In cases of extremity reconstruction, end‐to‐side arterial anastomosis is preferred to preserve distal perfusion. Two venous anastomoses are performed whenever possible. Typically, the deep venous system is more reliable in patients with XAWs (Fallahi et al. 2025). Patients were admitted for a median 7 days post‐operatively, both for flap monitoring and substance use rehabilitation.

Restoration of extremity function following soft tissue reconstruction often requires additional procedures, including tendon transfers, functional muscle transfers, and orthopedic reconstruction. Re‐elevation of the free flap should be anticipated. Patients presenting with an upper extremity or hand wound typically have involvement of the extensor mechanism, resulting in a contracture. Patients may require free vascularized bone grafting depending on the extent of bone debrided. One patient underwent a free medial femoral condyle flap outside of our study period to restore forearm stability. In addition, patients often undergo flap debulking via direct excision or liposuction to harmonize the soft tissue contour. Secondary procedures are performed at least 6 months from the date of soft tissue reconstruction to allow complete healing and ensure the patient can comply with the recovery process.

Pain management is challenging in patients undergoing XAW reconstruction. Patients should receive a pre‐operative nerve block or catheter to assist with post‐operative pain control when possible and be followed by an acute pain specialist. In all cases, addiction medicine should be consulted to assist in treating substance use withdrawal and managing a multimodal pain regimen in the setting of opioid tolerance. Suzetrigine, a novel non‐opioid pain medication, may prove particularly beneficial in this population. Unfortunately, elopement is common among patients with XAWs due to substance withdrawal (Johnsen et al. 2025), as we observed with two patients. Collaboration with social workers is critical to facilitating a safe discharge, preferably to an inpatient substance use rehabilitation center capable of providing any necessary wound care or a supportive home environment with involved caregivers. If a patient discharges AMA following free flap reconstruction, they should be educated on the signs of flap compromise and be given a clear plan to return to the hospital if there are concerns.

Continued xylazine use is a major risk factor for reconstructive failure and limb loss. Two of the five patients in our series (40%) who continued to inject xylazine at the recipient site experienced late wound recurrences observed on post‐operative days 388 and 300 after free flap reconstruction. These two patients ultimately underwent trans‐humeral amputation (Figure 2). To maximize the likelihood of success, substance use rehabilitation is necessary prior to free tissue transfer. Abstinence for at least 3 months is advisable, as this defines the early remission period in the DSM‐5. Patient‐specific factors, however, influence the ultimate timing of reconstruction. Exposure of critical structures, such as the airway, requires urgent attention. In addition, evaluation of a patient's housing and social support system is needed to determine their ability to recover from surgery. Nearly all patients in our series reported reliable housing at the time of surgery.

Our approach to XAW reconstruction fundamentally differs from that of other soft tissue wounds (Johnsen et al. 2025; Retrouvey et al. 2024; Makhoul et al. 2026). XAWs are a manifestation of the broader disease of addiction and must be managed in a multidisciplinary framework. Our pathway begins with involving addiction medicine consultants and wound care specialists for all cases of XAWs (Makhoul et al. 2026). If the XAW has resulted in a non‐functional extremity (e.g., no motor function due to nerve destruction), amputation is discussed. If present, acute infection is treated with serial debridement and antibiotics, with consultation from infectious disease colleagues. Once a patient has been abstinent from injection drug use for at least 3 months, we consider elective reconstruction through a staged approach. If the patient presents with a life‐threatening XAW, such as a neck or chest wound, we proceed with coverage of vital structures regardless of abstinence status. Our addiction medicine and social work colleagues are key partners in the ethical discussion of whether a patient has been adequately optimized for reconstruction, balancing the risk of recidivism and associated reconstructive failure.

This retrospective study has limitations. Xylazine toxicology testing is not routinely performed in our institution due to the high prevalence of xylazine in the illicit drug supply greater than 90% and a lack of clinical relevance (Drug Checking Quarterly Report (Q3 2022) 2022). Therefore, the diagnosis of a xylazine‐associated wound is made clinically based on history, physical exam, and opioid drug screening. In addition, our available sample size limited our ability to make statistically significant comparisons, though this is the largest series of free flap reconstructions for XAWs reported to date. Future research should focus on the timing of free flap reconstruction and period of abstinence required to predict a successful outcome. In addition, study of the quality‐of‐life changes following various reconstructive modalities such as skin grafting, free flap, and amputation is warranted.

## 5 Conclusions

Free tissue transfer is an effective reconstructive option for patients with exposed bone or critical structures in the setting of a xylazine‐associated wound and is necessary for limb salvage. Affected patients are typically young and benefit considerably from reconstruction. Late wound recurrence due to continued injection drug use is associated with flap failure and often results in limb loss. To optimize the likelihood of success and maintain reconstructive options, a multidisciplinary treatment approach and abstinence from injection drug use are critical.

## Author Contributions

Conceptualization: A.T.M., L.S.L., I.C.L., S.J.K. Methodology: A.T.M., C.Z.M., E.B.C., M.A.G. Formal analysis: A.T.M., C.Z.M., E.B.C., M.A.G. Supervision: J.B.M., L.S.L., J.D.W., J.P.F., I.C.L., S.J.K. Writing: A.T.M., C.Z.M., E.B.C., M.A.G., J.B.M., L.S.L., J.D.W., J.F.P., I.C.L., S.J.K.

## Funding

The authors have nothing to report.

## Ethics Statement

Exemption for this research was granted by the University of Pennsylvania Institutional Review Board #855779.

## Conflicts of Interest

The authors declare no conflicts of interest.

## Data Availability

The data that support the findings of this study are available from the corresponding author upon reasonable request.
